# Periscope of Dermatology

**Published:** 1899-05-06

**Authors:** 


### Periscope of Dermatology.

**Lupus, &c.** (*continued from p.* 80).—Boock[16] strongly urges the tubercular nature of lupus erythematosus, since all his fatal cases died of undoubted tuberculosis. He arrives at the following conclusions: (1) That lupus erythematosus is an eruptive inflammatory disease, its special localisation being determined by the vaso-motor centres, and it never is merely a local process; (2) it has a tubercular origin; (3) it is caused by the toxins of the tubercle bacillus; (4) the pathological changes are successively dilatation of the vessels, intoxication of the tissue cells, and secondary inflammation, the whole resulting usually in atrophy, but sometimes in necrosis. Unna[17] does not admit any connection between this disease and tubercle. In treatment he considers that one ought to avoid any remedy which might produce hyperæmia or œdema. He does not attach much importance to internal remedies, but believes that cases can be cured by external means alone. Among the most suitable applications are the following: (1) ℞. Zinc oxid, boli rubræ, a.a. 2 parts; boli albæ, mag. carb., a.a. 3 parts; amyli, 10 parts; fiat pulv. (2) ℞. Saponis viridis, 2 to 4 parts; collodium, 20 parts, M. On the whole he considers that alkalis and soaps are the most suitable applications. Whitehouse[18] records a case of lupus erythematosus which had resisted external applications, and was practically cured within three months after the administration of iodoform internally in one grain doses. Within a few days of the commencement of this treatment the lesions assumed an angry appearance; at the end of the third week the redness and inflammation began to subside, and at the end of a month the inflammation and most of the lupus had disappeared. No unpleasant constitutional effects followed the treatment.

**Erysipelas.**—Freeman[19] states that the streptococcus occupies, at first, exclusively the lymph spaces and vessels. Definite changes are produced in the blood; thus in the cutis the toxins act on the blood by forming rapid coagulation and consequent thrombi; there is also considerable leucocyctosis. In the subcutaneous tissue the disease is apt to become fibro-purulent, owing to the accumulaof leucocytes. Rosenberg[20] records the case of a man, aged 50, who developed erysipelas of the right eyelid, the inflammation spreading therefrom to the face, the neck, and finally over nearly the whole body. Gangrene of the eyelids occurred, but the patient recovered. The treatment adopted was the application of a solution of perchloride of mercury, followed by ichthyol vaseline, and finally subcutaneous injections of a 2 per cent. carbolic acid solution, together with camphor internally. Koetzen[21] relates an investigation into the use of metacresolantol in the treatment of experimental erysipelas in animals and of ordinary erysipelas in man. The disease was produced by the injection of the streptococcus, and treatment was commenced as soon as a florid rash developed. Painting the surface with the drug was found to be more efficacious than hypodermic injection. When the painting was carried out every two hours the results were very satisfactory, and no micro-organisms were found in the tissues after cure by painting. The treatment was also applied to five cases of erysipelas in man. The patch was painted for from 10 to 20 minutes every two hours for three or four days, and all the cases were cured. A 3 per cent. solution was used.

**Herpes, &c.**—Aitken[22] describes a case of herpes zoster affecting the leg below the knee. The eruption followed the course of the internal saphena nerve. Alger[23] states that it is best to open the vesicles in herpes, and then apply a solution containing picric acid, ʒ1½; citric acid, ʒ3; in ℥2 of distilled water. Severe neuralgic pain is best relieved by galvanism. Pardee[24] describes two cases of herpes iris, and discusses the pathology of the disease. The condition consists in an acute inflammation of the upper half of the corium, with dilatation of the superficial network of blood vessels, and lymphatics, the vesicles being formed by the lifting up of the entire epidermis from the papillary body, the contents of the vesicles being coagulated serum and leucocytes. The disease is very intractable. Brocq[25] reports a case of dermatitis herpetiformis with erythematous, vesicular, and bullous lesions, lasting a year and a half, in which the affected parts gradually took on a cicatricial aspect, and small epidermic cysts appeared similar to those described by Hallopeau as occurring in congenital bullous dermatitis. The application of static electricity seemed to exert a general tonic effect, and allayed the itching; a similar result was obtained after the application of electricity in a case of pruritus. Bowen[26] records a case of bullous dermatitis with epidermic cysts, in an anæmic girl, aged 12 years. The disease had first been noticed at the age of 3 weeks, and consisted of successive eruptions of bullæ, chiefly on the extensor surfaces and on the wrists. On the back of the hands there were firm whitish nodules, which yielded a soft cheesy substance when squeezed. The bullæ were often produced by some slight injury. Hallopeau,[27] in a further paper on this subject, divides congenital bullous dermatitis into three classes—viz., (1) a simple bullous form; (2) a bullous and dystrophic form, accompanied by atrophy of the skin and dystrophy of the nails; and (3) a deceptive form, where the bullous eruption has ceased to appear, while evidence remains of the disease in the scarring and dystrophy of the nails. Shillitoe[28] records a case of acute pemphigus occurring in a girl aged 19. The eruption was of an extremely severe type, but was cured by the local application of iodine lotion (1 in 5,000) and of liquor carbonis detergens. A diplococcus, agreeing with that found by Demme, was cultivated from the clear fluid of the bullæ. There was no clear history of any infection, but the patient may have been infected by meat handled in cooking. Hellier[29] reports a case of pemphigus foliaceus in the new-born. The mother was healthy, and there was no history of syphilis in either parent. When the child was nine days old, the nates and hips became unduly red; and on the tenth day large flaccid bullæ formed over the hips, abdomen, and back; much of the skin became denuded, leaving a large raw surface. The child looked as though she had been badly scalded. On the eleventh day the condition was rapidly spreading; there was a huge bulla on the left shoulder, and most of the skin was involved except the front of the chest. The child died on the fourth day of the disease, but the mother made an excellent recovery without any infective symptoms.

**Leprosy.**—Chapin[30] records four cases of leprosy treated with injections of the mixed toxins of erysipelas. There was not any benefit in any case, but no bad effects were noted. Sprecnck[31] has found that the serum of non-leprous people reacts to a cultivation of Hansard's bacillus in the same way as does the serum of non-typhoid patients to cultures of Eberth's bacillus; that is to say, the agglutination is imperfect or slight. He, therefore, suggests that a sero-diagnostic test resembling Widal's will be of service in doubtful cases. Von During[32] mentions two cases of leprosy in which direct contagion took place. (1) A young girl lived among leprous people for some years, and then contracted the disease; and (2) a man contracted leprosy from his wife. He, therefore, considers leprosy as a contagious infectious disease, but fortunately one which is not readily communicated.

**Various.**—Shield[33] records a case of multiple growths of the skin caused by exposure to the sun. The patient was a man, aged 35, whose occupation exposed him to the fierce rays of a South African sun. For about eight years he had suffered from growths on the face, and several of these had been removed from time to time, but they had returned; and when first seen by the author he had numerous growths, varying in size from a shot to a hazel-nut, studded about the skin of the face and the neck. They were removed as freely as possible, and on microscopic examination proved to be spheroidal-celled carcinomata, which had probably originated in the sweat glands.

Barski[34] reports a case of acanthosis nigricans occurring in a boy aged 13; it had commenced at the age of two. The symptoms are: Extreme pigmentation of the skin, hypertrophy of the papillæ, changes in the mucous membranes, nails, and hairs. Usually some disturbance of the general nutrition is also present. The pigmentation is always found on the neck, around the arms, external genitalia, also in the axillæ, navel, hands, elbows, breast, and about the anus. The affection is symmetrical. The hypertrophy of the papillæ is either slight and diffused, or very marked and localised, often reaching the formation of tumours. This hypertrophy also affects the mucous membranes of the nose, lips, gums, cheeks, and especially the tongue. The hair becomes dry and brittle, falling out easily; the nails become thick and brittle. Cachexia soon develops, accompanied by severe pains all over the body, especially the stomach. In some cases a carcinomatous growth can be discovered in the abdominal cavity.

[16] Brit. Jour. of Derm., Oct., 1898, p. 373. [17] Int. Med. Mag., Oct., 1898, p. 713; Jour. of Cut. and Genito-urinary Diseases, Oct., 1898. [18] New York Med. Jour., Feb. 11, 1899, p. 159. [19] New York Med. Jour., Feb. 4, 1899, p. 175. [20] Brit. Jour. of Derm., Nov., 1898, p. 423. [21] Brit. Med. Jour., Epit., Nov. 26, 1898, No. 411; Deutsch Med. Woch, Oct. 27, 1898. [22] Brit. Med. Jour., Mar., 1899, p. 712. [23] Med. News, New York, Aug. 27, 1898, p. 260. [24] Johns Hopkins Hosp. Bull., July, 1898, p. 165. [25] Amer. Jour. Med. Sci., Jan., 1899, p. 113; Ann. of Derm. and Syph., 1898, No. 7. [26] Glas. Med. Jour., Oct., 1898, p. 317; Jour. of Cut. and Genito-urinary Diseases, June, 1898. [27] Brit. Jour. of Derm., Jan., 1899, p. 44; Ann. de Derm. et Syph., Sept., 1898, p. 721. [28] Lancet, Nov. 26, 1898, p. 137. [29] Brit. Jour. of Derm., Jan., 1899, p. 18; Glas. Med. Jour., Feb., 1899, p. 155. [30] Med. Rev., New York, Jan. 7, 1899, p. 1. [31] Med. Rev., New York, Jan. 28, 1899, p. 132. [32] Brit. Jour. of Derm., Nov., 1898, p. 425; Deutsch Med. Woch., Nos. 20 and 21, 1898. [33] Lancet, Jan. 7, 1899, p. 22. [34] Int. Med. Mag., Jan., 1899, p. 45.

